# Ensemble and Greedy Approach for the Reconstruction of Large Gene Co-Expression Networks

**DOI:** 10.3390/e21121139

**Published:** 2019-11-21

**Authors:** Francisco Gómez-Vela, Fernando M. Delgado-Chaves, Domingo S. Rodríguez-Baena, Miguel García-Torres, Federico Divina

**Affiliations:** 1 Computer Science Division, Pablo de Olavide University, ES-41013 Seville, Spain; dsrodbae@upo.es (D.S.R.-B.); mgarciat@upo.es (M.G.-T.); fdiv@upo.es (F.D.); 2 Faculty of Experimental Sciences, Pablo de Olavide University, ES-41013 Seville, Spain; fmdelcha@alu.upo.es

**Keywords:** gene networks, scale-free networks, ensemble networks, graph theory, computational biology, mutual information networks, biomarkers discovery

## Abstract

Gene networks have become a powerful tool in the comprehensive analysis of gene expression. Due to the increasing amount of available data, computational methods for networks generation must deal with the so-called curse of dimensionality in the quest for the reliability of the obtained results. In this context, ensemble strategies have significantly improved the precision of results by combining different measures or methods. On the other hand, structure optimization techniques are also important in the reduction of the size of the networks, not only improving their topology but also keeping a positive prediction ratio. In this work, we present Ensemble and Greedy networks (EnGNet), a novel two-step method for gene networks inference. First, EnGNet uses an ensemble strategy for co-expression networks generation. Second, a greedy algorithm optimizes both the size and the topological features of the network. Not only do achieved results show that this method is able to obtain reliable networks, but also that it significantly improves topological features. Moreover, the usefulness of the method is proven by an application to a human dataset on post-traumatic stress disorder, revealing an innate immunity-mediated response to this pathology. These results are indicative of the method’s potential in the field of biomarkers discovery and characterization.

## 1. Introduction

Arising at the beginning of the century, Gene Networks (GN) have become a breakthrough in the analysis of biological processes in most gene expression studies. Such networks represent relationships between genes (or gene products) by means of a graph composed of nodes and edges, where nodes represent genes and edges the relationships among them. GNs have been widely used in both basic and applied research, such as biology [1], medicine [2], and diagnostics [3], among others.

GNs models also pave the way for hypotheses-making, which can be empirically validated afterwards. The results show significant reliability of GNs in this sense, since many predicted interactions have been experimentally confirmed later [4]. Therefore, algorithms and computational methods for GNs reconstruction have gained relevance among the Bioinformatics community [5]. These methods usually take gene expression datasets as inputs, e.g., microarrays or RNA-Seq data, for the inference of gene–gene relationships. To a greater extent, the vast amount of genetic information generated in the last decade has allowed the inference of relationships among DNAs, RNAs, proteins and other cellular components [6,7].

In this context, it is possible to classify GNs according to the inference approach used, including Bayesian, information theory, Boolean, or differential equations models, among others [8]. Consistently with this classification, co-expression networks, which are based on information theory, appear as a remarkably relevant approach due to their computational simplicity and low computational demands [9]. These networks infer relationships between genes that show similar patterns of expression. This is achieved by measuring the degree of relationship between each pair of genes, so the relationship is only approved when this degree exceeds a certain threshold. This threshold value indicates the minimum level of similarity between two expression patterns for the relationship to be considered significant. Therefore, the higher this threshold is, the sparser inferred GNs will be [10]. According to the published literature, the main measures to evaluate the co-expression degree between two genes are correlation measures such as Pearson, Spearman or Kendall coefficients [11,12]. Additionally, other measures have been widely used for the generation of GNs such as Mutual information [13].

Nevertheless, co-expression networks usually present two main drawbacks: (a) the above-mentioned measures present some limitations [14], for example, their inability to detect non-linear dependencies or their dependence on the distribution of the data, as in the case of Spearman and Pearson coefficients, respectively [15]; and (b) inferred networks are often too densely-connected to perform comprehensive analyses, being actual GNs known to be sparse [16].

As far as the topology of the networks inferred is concerned, GNs should generally meet a series of requirements. First, GNs should follow a scale-free topology, as they have been proven to be sparse [17,18]. Thus far, scale-free GNs reconstruction entails a major challenge as algorithms themselves show limitations in distinguishing truly-significant interactions, thus providing densely-connected networks. Second, it is to be highlighted that biological networks contain hubs, which are genes influenced by a significant number of relationships. Hubs are then key elements in the control and regulation of the genes comprised in the network, and have proven their importance in the modeling and analysis of genetic interactions [19,20,21]. It follows that inferred GNs should contain hubs. As consequence of these two requirements, GNs topology optimization arises as a major issue to be faced.

In this work, we propose a novel approach for the reconstruction of large gene co-expression networks. In particular, we propose a two steps strategy to induce gene networks. In a first phase, an ensemble approach is used in order to generate co-expression networks. The so-obtained network is then optimized in a second stage, where a greedy strategy optimizes both the size and the topological features of the network.

Not only is this method able to overcome the limitations of using a single measure to assess gene co-expression thanks to an ensemble strategy, it also carries out a greedy heuristic topological optimization of the inferred GNs. Therefore, we can summarize our contributions as follows:

- The method is able to overcome the limitations of a single information theory measure thanks to an ensemble strategy.
- The method is also able to perform a topology optimization.
- The experiments carried out show that our approach achieved good results against other state of the art methods.
- The usefulness of the proposed method becomes evident in an application to a study of a post traumatic stress disorder on human dataset.
- The method’s results show its potential in the field of biomarkers discovery and characterization.

### Related Work

Co-expression analysis assumes that genes whose mRNAs show similar level of variation upon perturbations are involved in the same, or closely related, biological processes. Approaches based on such assumption haven been considered as promising for the discovery of genes implicated in biological processes of interest [22]. Particularly, co-expression networks have provided valuable insights on diseases’ underlying molecular mechanisms, as in the case of cancer [23].

In Reference [24], weighted gene co-expression networks were analyzed to investigate the role of gene regulation in lung cancer. Using Pearson correlation coefficients for gene pairs, the authors detected a lung cancer-specific module of co-expressed genes with clear functional interpretations. Pearson’s measure, and the Weighted Gene Co-expression Network Analysis (WGCNA) methodology [25], were also used by Ivliev et al. [26] to identify gene co-expression modules covering a range of known tumour features. The WGCNA methodology implies not only taking into account the correlation between a gene pair, but also whether these genes are correlated with similar sets of genes across the entire transcriptome. Other works use different co-expression measures. For example, Yuejie et al. [27] assumed that two genes that use the same dictionary to represent their original expression values must share similar co-expression patterns. In this case, the authors used a sparse coding and dictionary learning algorithms.

Despite the good results achieved in previous approaches, the measures used present some limitations, as mentioned in the previous section. Thus, recent works have been focused on the possibility of combining different inference methods and co-expression measures. For example, in [28], an Ensemble-based Network Aggregation method (ENA) is proposed to integrate gene networks derived from different methods and datasets, in order to improve the accuracy of network inference. Other works try to combine different pre-processing methods (see, e.g., [29]). In this work, the network inference problem between *g* genes is decomposed as *g* separate regression problems. Thus, an ensemble of several feature selection algorithms are used to find those genes most suitable in modeling the expression values of every target gene. Besides looking for the best co-expression measure, other studies try to use different inference methods. In [30], three normalization methods and 10 inference methods, including six correlation and four mutual information methods, were tested. Liue et al. [31] presented a novel inference algorithm, namely Local Bayesian Network (LBN). This algorithm applies an iterative methodology, in which, firstly, conditional mutual information is used to generate an initial network. Then, it uses a *k*-nearest neighbor approach to decompose the network into smaller sub-networks. Finally, the algorithm identifies and removes redundant relationships between genes using a Bayesian method. These new sub-network are integrated into a new gene network and the process restarts until the topological structure of the network remains unchanged.

In addition, the optimization of gene co-expression networks represents a challenge due to the size and complexity of the data from which the networks are obtained. Hence, the goal is to reduce both size and complexity of the final network while maintaining biological relevance. Network structure optimization is a NP-hard problem, so some works use heuristic algorithms to explore the possible combinations of all interactions in order to simplify the network structure [32]. However, these approaches usually present computational limitations due to the high dimensionality of the networks [33]. Other works use evolutionary techniques to reduce the large search spaces. For example, in [34], a genetic algorithm is used to reconstruct gene networks from time-series expression profiles based on fuzzy cognitive maps. Some research works based their optimization efforts on objective functions and scores (see, e.g., [35]). In this work, an undirected confidence-weighted likelihood matrix is created using pairwise confidence scores from functional association databases. Using this matrix, GNs are inferred with a high accuracy level. Other researchers, e.g., Lopes et al. [36], use a scale-free topology information to prune search space during inference problem. Finally, in the research presented by Yang et al. [37], a bayesian-based inference process is used to evaluate the relative importance of nodes.

## 2. Materials and Methods

In this section, we present the different methods and datasets used in this paper. In particular, Section 2.1 describes the proposed method for large GNs reconstruction, while, in Section 2.2, we describe the datasets used in the experiments. Finally, Section 2.3 introduces the measures used to assess the performance of the method.

### 2.1. EnGNet: Gene Network Reconstruction Based on Ensemble Strategy and Greedy Optimization

**Figure 1:**
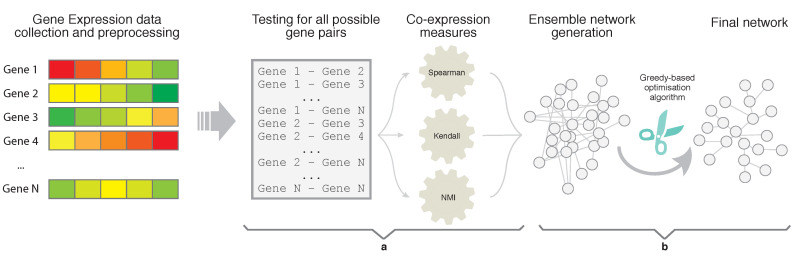
Global workflow of EnGNet for GNs reconstruction. As shown, the method is based on two different steps: (**a**) an ensemble strategy for network inference; and (**b**) a greedy-based approach for the final optimization (maximum spanning tree algorithm).

In this section, we introduce the proposed method for large co-expression networks generation, which we name Ensemble and Greedy networks (EnGNet). A EnGNet JAVA-based implementation is available at: https://github.com/fgomezvela/EnGNet (accessed on 15 November 2019). As previously introduced, EnGNet comprises two main steps, described in Figure 1: (a) an ensemble-based method to infer gene–gene co-expression relationships; and (b) a greedy strategy for the topological optimization of the network. As a result, the final network exhibits not only reliable interactions but also lower topological complexity and sparseness than other techniques that adopt single co-expression measurements. As stated in Section 1, the spareness in a GN is a desirable feature, involving a significant improvement over other methodologies.

#### 2.1.1. Ensemble Strategy for Network Generation

In the first phase, EnGNet induces a single co-expression network, using three different evaluation measures. In this case, three widely-used co-expression measures were selected for assessing the significance of gene–gene interactions. In particular, we used the Spearman, Kendall coefficients and Normalized Mutual Information (NMI) measures. Our choice is motivated by the following observations. The Spearman coefficient is able to detect linear dependencies between two genes unaffected by data distribution. Kendall’s measure is also able to detect linear dependencies but has advantages over Spearman’s in approaching distribution normality more rapidly [15]. Finally, the NMI is able to detect linear and also non-linear dependencies between genes [38].

The three measures used provide a value $v_{i}$, $0\leq v_{i}\leq 1$, where 0 represents no dependency and 1 a total dependency between the genes.

**Figure 2:**
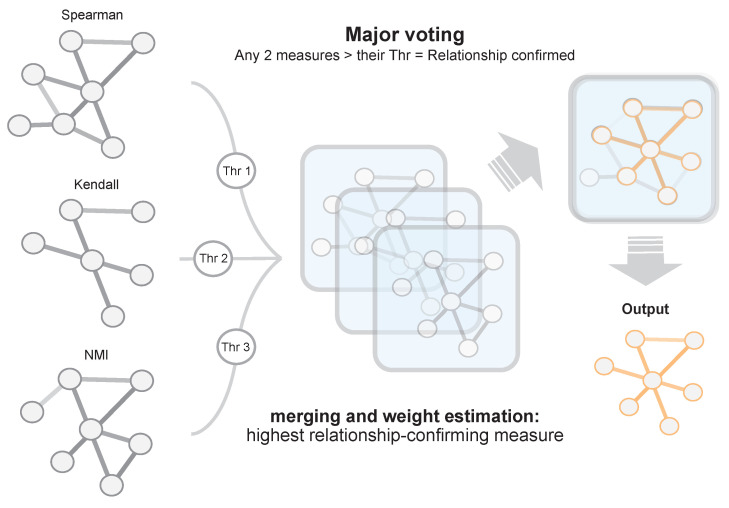
Schematic representation of the Ensemble step of EnGNet. Three well-known measures for the generation of co-expression networks are combined, here by Spearman, Kendall and NMI, by means of an ensemble strategy.

The reconstruction process is based on the evaluation of all possible gene pairs. As shown in Figure 2, the three measures are used for evaluating every gene pair relationship. For each measure, a significance threshold ($Th_{i},1\leq i\leq 3$) is used in order to determine whether or not the relationship is considered valid by a specific measure.

The final significance assessment is carried out through a voting system. Thus, a relationship is confirmed if it is considered significant by at least two measures (see Table 1).

Hence, a relationship is added to the final network if it is considered correct, and its final weight, denoted as $w_{en}$, is set to the average value $v_{i}$ of the three measures. Doing so, we subsume the information of the three measures in a single value. The so-created network represent the input to the second step of the proposed strategy.

#### 2.1.2. Topological Optimization Based on Greedy MST Algorithm

**Figure 3:**
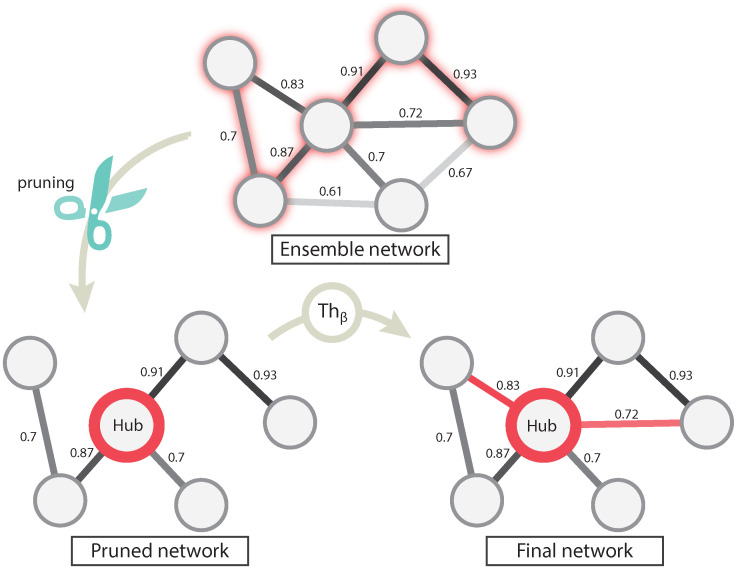
Graphical description of the second step of EnGNet. First the previously-obtained ensemble network is pruned by a MST (minimum spanning tree)greedy algorithm. In a second phase, the most relevant edges, which were initially pruned, are evaluated with a threshold ($Th_{\beta }$), and added to the final network again.

In this step, the topological features of the network obtained in the first step described in previous section are optimized by means of two phases: pruning and adding relevant edges (see Figure 3). In the first phase, the ensemble network is pruned using a greedy-based heuristic algorithm, which takes into account the most relevant interactions, i.e., those showing the highest co-expression weight according to ensemble step. In particular, we used the modification of the Kruskal’s minimum spanning tree (MST) algorithm presented in [7] to obtain the longest path between each pair of genes. This modification consists of selecting the most significant edges, instead the less relevant ones, until all nodes are connected with no cycles. Thanks to this, the method obtains the most significant path between each pair of nodes that comprising the network [39].

As a result, the method computes a pruned network (see “Pruned network” in Figure 3), which contains the same number of nodes as the original network, albeit keeping only most relevant relationships. This reduction in edges significantly improves the sparseness of the network.

However, not all removed relationships are necessarily irrelevant to the network. For this reason, in the second phase, a topological analysis of the pruned network is performed in order to identify network’s hubs. As stated in Section 1, hubs play a crucial role in how the information is distributed through the network and usually these are key regulators of the genes involved. For this reason, hubs are selected as those nodes whose connection degree exceeds the average network connectivity (see “Pruned network” in Figure 3 where the hub is highlighted as the node showing the greatest number of relationships).

Once the hubs have been identified upon the pruning process, they are independently processed. For each hub, its linking edges that were removed in the ensemble network are again evaluated using a threshold $Th_{\beta }$. This threshold is a user set parameter, which is employed to determine the biological relevance level of the removed edges. Each individual edge will be added to the network if its weight $w_{en}$ (calculated in the ensemble step) exceeds $Th_{\beta }$. Note edges are not recalculated as they are preserved from the first step (Section 2.1.1).

Note that, after pruning, those nodes exceeding the average node’s degree are selected as potential hubs. In addition, the pruning step drastically reduces the average node degree. After the second step, where edges are added using the threshold $Th_{\beta }$, hubs are enriched in edges so these greatly exceed the average network connectivity. The final network generated by EnGNet is obtained after this step (see Figure 3).

A complete pseudo-code of EnGNet is described in Algorithm 1 and 2.

**Algorithm 1:** A general pseudocode of EnGNet. The method is divided into two different steps: (a) the ensemble network generation; and (b) a structure optimization by means of MTS algorithm. The pseudocode of the function *ensembleEdge* is given in Algorithm 2

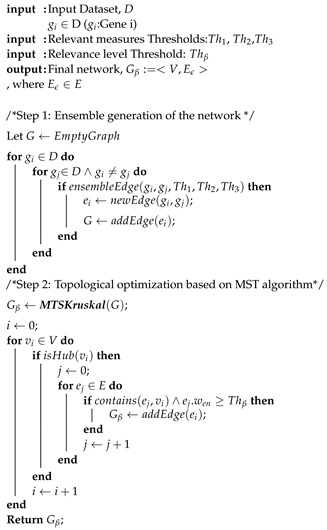



**Algorithm 2:** A general pseudocode of *ensembleEdge* function.

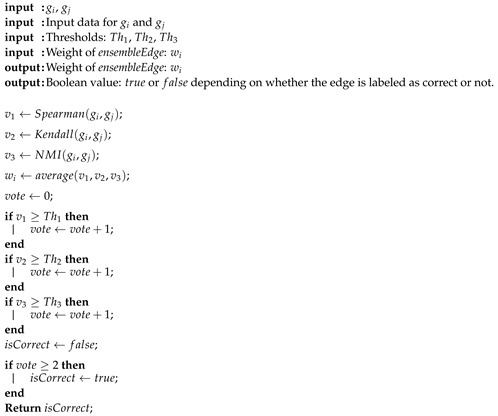



### 2.2. Datasets

In this section, the datasets used to test the usefulness of the proposed method are described. To this aim, we selected three datasets related to two different organisms that present different features: *Saccharomyces cerevisiae* and *Homo sapiens*. These organisms represent evolutionary-distant species, showing increasing complexity.

*Saccharomyces cerevisiae***cell cycle dataset** The dataset presented by Spellman et al. [40] was selected, which has been widely used for gene networks inference. This dataset contains the information about yeast cell cycle-related genes through a microarray analysis of the expression level of 5521 genes. RNA samples were collected from yeast cultures, which were synchronized by means of three different methods: α factor arrest, elutriation, and cdc15 thermosensible mutant.
*Homo sapiens***SNP dataset** The first selected human dataset, which was presented by Hodo et al. [41], was used in a study of the associations between interleukin 28B SNPs and recurrence of hepatocellular carcinoma (HCC) in patients with chronic hepatitis C (CHC). For the original purpose, the effects of a certain IL-28B genotype were tested by comparison of microarray data of 20 HCC patients vs. 91 CHC patients. The mentioned dataset stores expression levels of 54,616 human genes.
*Homo sapiens***Post-Traumatic Stress Disorder (PTSD)** Finally, a dataset testing PTSD, presented in the work by Breen et al. [42], was selected. This dataset was obtained to compare lymphocytic gene expression levels between PTSD-diagnosed US marines and control cases. Samples were collected from 94 marines (47 cases and 47 controls) both previously and subsequently to battlefield deployment. Thus, the dataset is divided into pre-deployment samples (controls) and post-deployment samples (cases). For the sake of simplicity, they are named “Pre” and “Post” for the rest of the paper. The dataset, harboring 27974 genes, were normalized as they comprise microarray (pre-deployment samples) and RNA-Seq (post-deployment samples) expression data. Additionally, this dataset was comprehensively analyzed to test the biological utility of the EnGNet tool in the experiment section.

### 2.3. Performance Evaluation of Gene Association Network

To assess the quality of our proposal, we present a comparison of the results obtained by EnGNet with those obtained from different methods from the literature on the datasets described in the previous section. To do so, we selected GeneMANIA [43] as the gold-standard to obtain different quality measures of the evaluated networks.

GeneMANIA is a gene interactions web-repository, which stores information presented in the form of web application for generating hypotheses about gene functions. It is possible to access online and freely the information stored in GeneMANIA. The genetic relationships identified in this database range from curated relationships that have been experimentally demonstrated to others that have been predicted in silico. A gene–gene relation is maintained in the database if at least one piece of evidence of such relationship exists in the literature. We selected GeneMANIA since it is a reliable source to test the correctness of gene–gene interactions [7,44,45], and it has demonstrated its suitability for this purpose in multiple previous works.

In this paper, the information stored for the two used organisms, i.e., *S. cerevisiae* and Human, was selected. The final networks obtained from GeneMANIA database are composed by 6462 nodes and 4,833,480 edges for yeast, and 19,551 nodes with 6,979,630 relationships for Human network.In particular, we based the comparison on two well known measures, namely precision and recall [9,15], which are defined as in the following equations:

(1) $$Precision=\frac{TP}{TP+FP}$$

(2) $$Recall=\frac{TP}{TP+FN}$$

where

- True positives (TP) is the number of edges contained in both the network obtained by EnGNet and in GeneMANIA.
- False positives (FP) is the number of edges that are contained in the network obtained by EnGNet but not in GeneMANIA.
- False negatives (FN) is the number of edges appearing in GeneMANIA but not in the network obtained by EnGNet.

### 2.4. Topological Features of Biological Networks

With the aim of evaluating the biological attributes of the networks that are presented (from a topological point of view), various criteria can be used. In the following, we present the most commonly used topological feature criteria of scale-free networks [6,46]:

**Average Clustering Coefficient:** Calculated as the number of edges linking nodes within its neighborhood divided by the number of links that are possible among them. A low clustering coefficient for a network is an indicator of the existence of biological relationships, as the lower this parameter, the sparser the network. Sparseness is also considered a main feature of GRNs.
**Characteristic Path Length (CPL):** Indicates the average length of the shortest paths between each pair of nodes comprising the network. A high path length indicates that the network is in a linear chain, while a lower value means that it is more compact. Scale-free networks usually have larger CPLs.
**Diameter:** Indicates the maximum distance between two nodes. As in the case of CPL, a high value indicates that the network follows a biological pattern.
**Graph Density:** Defines the ratio between the number of edges of a network and the number of all possible edges. Gene networks are generally sparsely connected so a low density is indicative of a biologically-meaningful pattern.
**Node Degree Distribution:** Defined as the number of edges linking a node. The larger is the degree. the more relevant is the node in a certain network. A distribution function P(k) defines the spread of node degrees over a network. This function represents the probability of finding a degree of *k* in a randomly-selected node. The degree distribution usually follows a power law of the form $P\left(k\right)∼k^{-\gamma }$, where γ is a constant (≥0). A high γ is indicative of a scale-free topology [47].

## 3. Results and Discussion

In this section, we present the results of the experimentation carried out in order to assess the reliability and usefulness of EnGNet. We first compared EnGNet with three standard information theory approaches commonly used in the literature to infer large GNs (based on NMI, Spearman and Kendall measures). Moreover, we compared our proposal with the ensemble strategy of these methods (i.e., only the first step of EnGNet). The aim of these experiments was to test the performance of EnGNet against other classical methods from the literature to infer large co-expression networks, and also to test the relevance of the prune step in the final results obtained. Thus, we not only tested the reliability of the inferred networks, but also the ability of EnGNet to reduce the size of final networks and their topological features.

In the second experiment, we also tested the performance of EnGNet against different algorithms from the literature for generating small gene networks. In particular, we present a study on 20 yeast genes that encode the Cell Cycle G1 phase.

Finally, with the aim of proving the effectiveness of our proposal in a biomedical study, we applied EnGNet to a human dataset regarding post traumatic stress disorder (PTSD).

### 3.1. Comparative Analysis Of EnGNet For Large Gene Networks

In the experiments, we used five approaches to generate networks from each dataset. In particular, we used EnGNet, the first phase of EnGNet, i.e., only the ensemble strategy without the pruning phase, and three information theory based methods. These last three methods are based on the NMI, Kendall and Spearman measures, in a similar way as the experiments presented in [7,15]. These approaches have been widely used in the biomedical literature for studying with gene co-expression networks (e.g., Xu et al. [48], Johnson et al. [49] and Liu et al. [50]).

For each information theory method used, we needed to set a validity threshold, and in the case of EnGNet, we needed four thresholds (see Section 2.1). For this experiment, we selected three different thresholds for all methods: 0.7, 0.8, 0.9. For a fair comparison, EnGNet and the ensemble approach also used the same thresholds for $Th_{1,2,3}$ and $Th_{\beta }$. These thresholds represent a complete full spectrum from a mid correlation (0.7) to a very strong one (0.9). Thus, 60 networks were generated and analyzed (5 methods × 3 thresholds × 4 datasets).

#### 3.1.1. Networks Performance Against GeneMANIA

As mentioned above, we first tested the biological significance of the obtained networks in a direct comparison with GeneMANIA database. The results obtained, in terms of nodes, edges, precision and recall, are presented in Table 2, Table 3, Table 4 and Table 5, respectively.

Table 2 shows how EnGNet achieved the second best results of the experiment (only behind Kendall’s) in terms of average precision. However, it is important to notice that EnGNet is the method that presents the most stable precision and size values for the different thresholds, obtaining the sparser networks for all methods considered (almost half the average size compared to Kendall’s). This result confirms the overall stability of EnGNet.

The experiment carried out on the Human SNP dataset shows that EnGNet obtains the best results in terms of average precision (see last row of Table 4). We can also notice that the NMI approach infers smaller networks than EnGNet. However, the precision is so low that these networks do not appear to be biologically significant.

For the experiments with “Pre” and “Post” PTSD datasets (Table 3 and Table 5, respectively), the results present the same pattern: EnGNet obtains the best results in term of precision and size of the networks.

**Figure 4:**
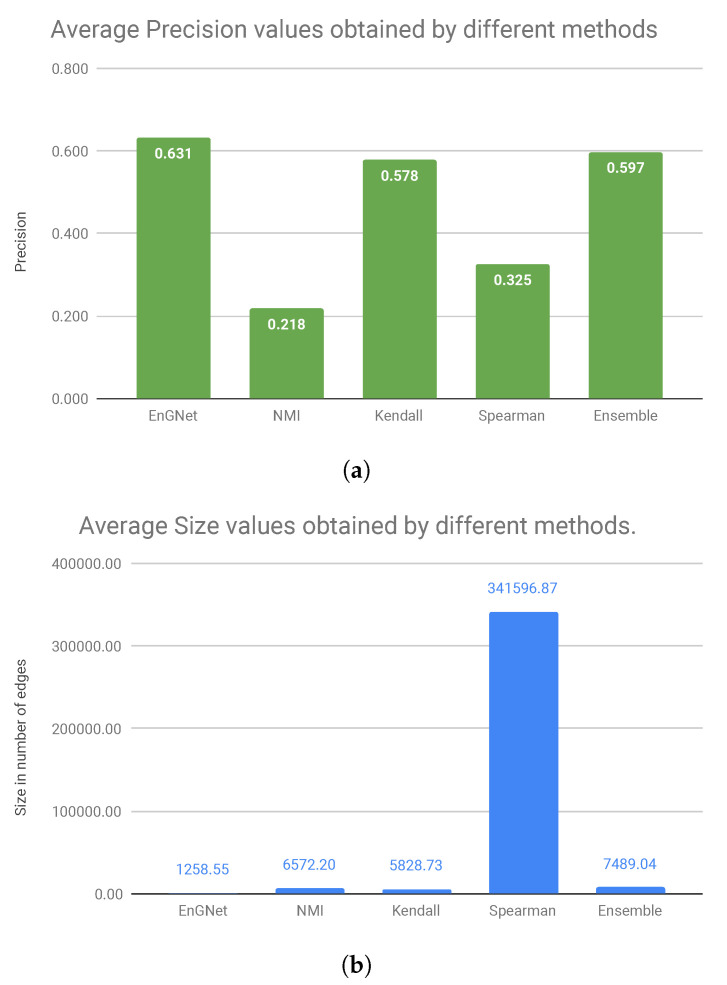
Visual comparison of the average results presented in the tables for all datasets. As it is possible to see in the chart, EnGNet obtains smaller networks with the best results in the precision experiments and the sparsest networks. As discussed above, these are desirable features for any method that infer large gene networks: (**a**) average precision values; and (**b**) average size of the networks.

Finally, Figure 4 shows the average values of precision and size of the networks for all experiments presented above. Considering the precision results presented in Figure 4a, we can observe that our algorithm is the one that obtains the best values, followed by the Ensemble approximation and Kendall’s. Regarding the size of the networks, it can be verified in Figure 4bthat EnGNet obtains the smallest networks (approximately 271 times smaller than Spearman’s network or six times smaller than Ensemble’s network, which is the second approximation in precision values) with the highest precision values.

In summary, we can conclude that EnGNet is successful in reducing the size of the networks while keeping competitive results in terms of precision and recall (against other methods studied). In fact, networks generated by EnGNet are significantly sparser than those obtained by other methods (see Figure 4). As stated above, this is a significant result, since sparseness is a desirable feature in GNs reconstruction from a large dataset. In fact, the smaller is the networks, the easier is their analysis [51]. Additionally, although networks are sparser in terms of the number of edges, precision and recall values do not suffer a relevant loss. This observation is confirmed from the results presented, since EnGNet obtains average precision values above 0.5 in all the cases studied (presented in the tables).

Finally, Figure 4 shows that EnGNet obtains the best average precision value, whilst the size of the network is significantly reduced (especially against the Spearman’s approach). This result indicates that EnGNet networks do not lose biological significance upon pruning. As a conclusion, we can affirm that EnGNet is a competitive and reliable method for the generation of large gene networks.

#### 3.1.2. Topological Features Analysis

In addition to network sparseness, the topological properties of gene networks should be considered in order to estimate the performance of EnGNet upon network reconstruction [7,9,16]. As discussed in Section 1, biological networks tend to be sparse and to follow a scale-free topology. Therefore, it is desirable for the reconstruction methods to provide networks that present such topological features.

With the aim of performing a topological analysis of the generated networks, we extracted the topological features presented in Section 2.4 for all networks discussed in Section 3.1. The results are shown in Table 6, Table 7, Table 8 and Table 9.

From these results, we can observe that EnGNet obtains the most stable results over the experiments carried with respect to the majority of the topological features studied (see “Average” rows in the tables). To clarify these results, we also calculated the average values for all datasets and thresholds presented. These results are reported in Table 10. In the table, it is possible to observe that, for all topological features studied, EnGNet is the algorithm achieving the best results, except for the network diameter. For the network’s diameter, only the Spearman’s method obtains better results. This is a logical result since Spearman’s method generates the biggest networks (271 times bigger than EnGNet). It is remarkable, from a topological point of view, that our method reaches a diameter in a similar range with a significantly smaller size than Spearman’s network.

In summary, EnGNet obtains the best results on all topological features, for all the networks, indicating that EnGNet networks follow a biological pattern (scale-free topology). Furthermore, EnGNet-generated networks improve the results obtained by information theory methods and ensemble networks. Bearing this in mind and the results presented in the comparison with the network contained in GeneMANIA, we can affirm that EnGNet is a suitable tool for large co-expression GNs reconstruction in biomedical research.

### 3.2. Comparative Analysis Of EnGNet For Small Networks

The ability of our approach to infer small gene networks was also tested. To do so, we performed a similar experiment to the one presented by Gallo et al. [52]. In this experiment, precision was used as quality measure to rate the reliability of the input GNs. The main objective of the experiment is to compare the precision values of different gene networks algorithms from the literature on the same dataset.

To obtain the input networks, we used different methods from the literature, which are described in the works by:

- Soinov et al. [53], a C4.5-based method;
- Bulashevska et al. [54], a Bayesian-based method;
- Ponzoni et al. [55], a combinatorial optimization algorithm (GRNCOP);
- Gallo et al. [52], an upgraded version of the previous algorithm named GRNCOP2; and
- Gomez-Vela et al. [15], a fuzzy method to infer gene co-expression networks named FyNe.

These methods were applied to the same dataset from the Yeast Cell Cycle—more specifically, to a subset of 20 well-described genes. These genes code for key proteins in cell-cycle regulation, as presented by Martinez-Ballesteros et al. [56].

As in the experiment performed by Gallo et al. [52], the quality of the networks was assessed regarding the precision values obtained against the data stored in YeastNet [57]. YeastNet is a repository that comprises a probabilistic functional GN generated from verified protein-coding open reading frames (ORFs) of the yeast genome. This repository combines protein–protein interactions, protein–DNA interactions, co-expression, phylogenetic conservation and literature information, in total covering more than 102,803 linkages among 5483 yeast proteins (95% of the validated proteome).

**Figure 5:**
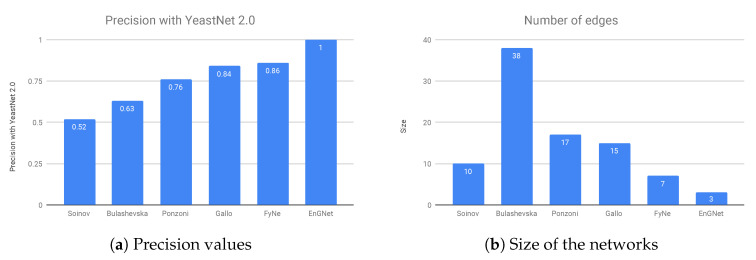
(**a**) Results from different methods on the 20 genes from the yeast cell cycle dataset. The results show that EnGNet is also a reliable method for inference of small co-expression networks with a high precision. (**b**) Size in terms of number of relationships. Note that EnGNet is again the method that obtain the smaller network.

The results of the experiment are presented in Figure 5a,b, where it can be verified that EnGNet yields the best results amongst all studied methods, and again with the smaller network. Note that the inference of small gene networks usually provides higher precision results than in the case of large ones, as detailed in Hecker et al. [16]. The results show that not only is EnGNet suitable for large gene networks studies, but also obtains competitive results for studies with small datasets.

### 3.3. Application to the Study of Human Post Traumatic Stress Disorder

The second objective of this study was to prove the usefulness of EnGNet in actual life sciences research. To do so, EnGNet was applied to a human PTSD dataset obtained by Breen et al. [42], so as to shed some light over the genes involved in this pathology.

In this case-control study, expression data were obtained from US marines peripheral blood leukocytes both before and after deployment to conflict zones (that called “Pre” and “Post”). As stated above, 94 marines (47 cases and 47 controls) were analyzed. According to the original article by Breen et al. [42], controls refer to selected marines who did not show signs of PTSD. These are used as a reference for cases, which are marines who show a broad spectrum of signs that classify them as under PTSD after battlefield deployment. PTSD was scored through a diagnostic interview and annotated in the Clinician Administered PTSD Scale (CAPS) [58]. In the experimental design, cases are analogous to controls prior to battlefield deployment, i.e., none are under PTSD symptomatology. On the other hand, after battlefield deployment cases significantly differ from controls in terms of the CAPS score (see the original article by Breen et al. [42] for further details).

**Figure 6:**
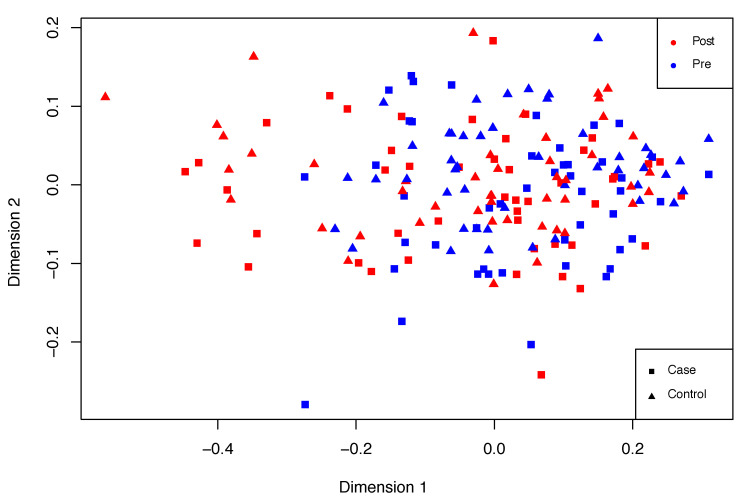
Non-supervised exploratory MDS plot showing differences between the input samples. RNA-seq (cases, squares in the figure) and microarray data (controls, triangles in the figure) were normalized and joined in a single dataset. Thus, no significant differences were expected between them. However, two groups for pre-deployment (red) and post-deployment (blue) are modestly differentiated, although cases in between are also appreciated.

Overall, PTSD signs may be observed in the second group when compared to the first one. An exploratory multidimensional scaling (MDS) plot or Principal Coordinates Analysis (PCoA) was performed in order to roughly examine these differences. MDS assisted the examination of sample similarity. On this occasion, the classical MDS method was applied, assuming Euclidean distances. An illustrative distribution of this dataset is shown in Figure 6, in which differences can be observed between post- and pre-deployment marines. However, these differences are fuzzy and there is a spectrum of sample states between pre- and post-deployment situations.

First, a differential gene expression analysis was carried out to verify the mentioned differences using the DESeq2 [59] R package, a tool for the estimation of differentially-expressed genes (DEGs). The information on gene up- or down-regulation was of especial interest in the analysis of the biological processes underlying PTSD development. Hence, data provided by DESeq2 were latter imported into Cytoscape for network interpretation purposes.

EnGNet was used to reconstruct two different networks corresponding to pre-deployment and post-deployment samples, respectively. To this aim, the EnGNet $Th_{1,2,3}$ thresholds were set to the values that yield the best results in the experimentation presented in Section 2.3, namely $Th_{1}$ = 0.7, $Th_{2}$ = 0.8 and $Th_{3}$ = 0.9. As far as the $Th_{\beta }$ threshold is concerned, a new analysis was carried out to determine the optimal threshold for each sample. The results of this study are presented in Table 11 and show the values of the precision and recall measure obtained by different networks against GeneMANIA.

Therefore, considering the results presented in the table, candidate networks for this study correspond to $Th_{\beta }$ = 0.8 in the pre-deployment case and $Th_{\beta }$ = 0.9 in the post-deployment situation.

**Figure A1:**
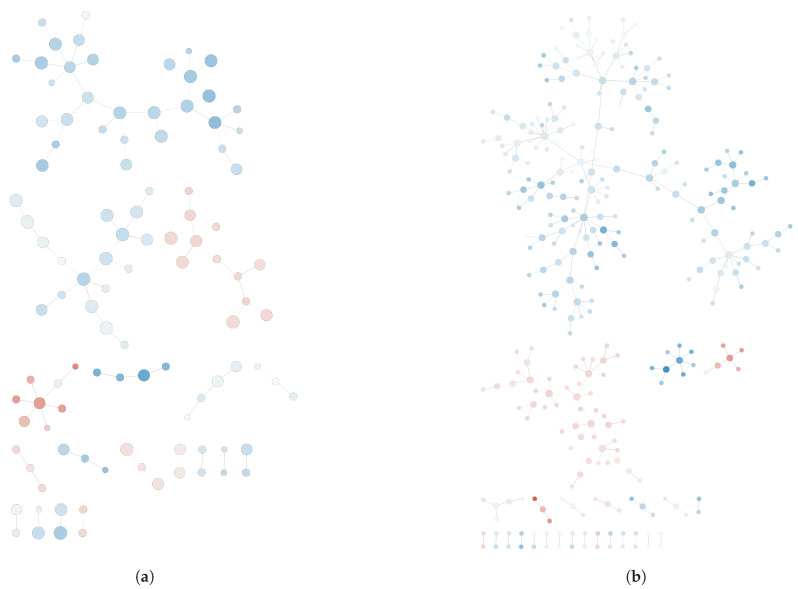
Inferred networks corresponding to: (**a**) pre-deployment samples; and (**b**) post-deployment samples. Log2 FC is represented by node color, so blue and red intensities are related to gene up- or down-regulation, respectively. Node size is represented according to their rank. Edge transparency is represented according to edge weight. Note both networks show a major connected module and exhibit a scale-free topology.

Once the networks were generated, a significant increase in the number of genes was found in the post-deployment network compared to its pre-deployment counterpart, which is indicative of gene up-regulation in lymphocytes upon PTSD development. Pre- and post-deployment networks are shown in Appendix A (see Figure A1). Remarkably, the reconstructed networks for pre-deployment and post-deployment samples were significantly different, which is indicative for the discrimination power of the GN reconstruction approach over other unsupervised techniques such as PCoA.

**Figure A2:**
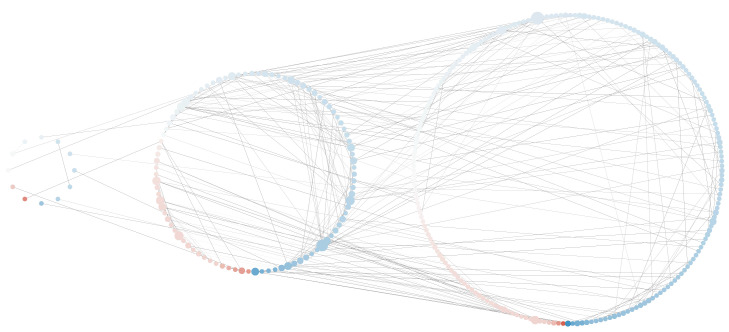
Union of pre- and post-deployment reconstructed networks. Nodes are sorted depending on whether they are exclusively present at the pre-deployment network (far left), exclusively present at the post-deployment network (far right) or present at both networks (center). Node size is represented according to their degree. Edges transparency is represented according to their weight. Upregulated and downregulated genes in post-deployment samples compared to pre-deployment samples are, respectively, shown in shades of blue and red.

Pre- and post-deployment networks were merged in order to graphically observe the differences in gene expression upon PTSD development. Overall, 73.8% of the nodes in this merged network were found to be upregulated in the post-deployment situation compared to pre-deployment, which suggest the importance of gene activation upon PTSD development. Genes up/down-regulation in the merged network is shown in Appendix A (see Figure A2).

Enrichment analysis was performed by means of Cytoscape’s plugins ClueGO [60] and CluePedia [61], which shows over-represented GO-terms in a ensemble of genes. ClueGO + CluePedia analyses provided useful information about the biological processes in which the genes comprised at the pre-deployment and post-deployment networks were involved.

**Figure 7:**
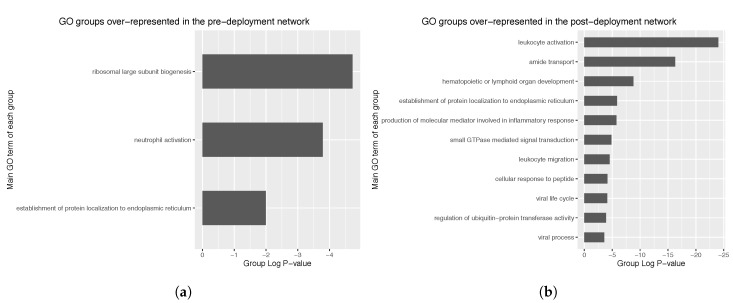
Bar plots showing the different groups of analogous GO terms that were identified in: (**a**) the pre-deployment network; and (**b**) the post-deployment network. The main GO term of each identified group, i.e., the one with lowest term P-value, is presented as group label. Group P-value was corrected with Bonferroni step-down. Note the lower is the P-value, the more the over-represented is the GO term.

Regarding the pre-deployment network (105 nodes), three different GO groups were identified, corresponding to ribosomal biogenesis, neutrophil activation and establishment of protein localization to endoplasmic reticulum (Figure 7a). Group *p*-values observed were of the order of 10^−6^.

**Figure 8:**
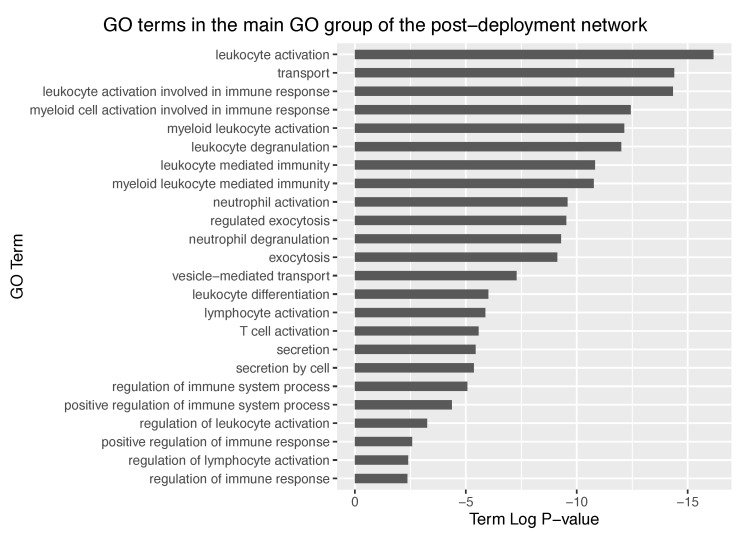
Top GO terms in the main GO group of the post-deployment network. Term P-value was corrected with Bonferroni step-down. Note the lower is the P-value, the more over-represented is the GO term.

In the case of the post-deployment network (298 nodes), 10 GO groups were identified, mostly corresponding to leukocyte activation, amide transport and hematopoietic or lymphoid organ development (Figure 7b). Observed group *P*-values were of the order of 10^−25^, thus representing a dramatic increase in significancy compared to the pre-deployment GO groups. Further exploration of the main GO group in the post-deployment revealed GO terms such as leukocyte activation involved in immune response, myeloid cell activation involved in immune response, myeloid leukocyte activation, and leukocyte degranulation. Main GO terms comprised in the main GO group of the post-deployment network are shown in Figure 8.

Enrichment analyses thus revealed a dramatically different situation in the post-deployment network compared to pre-deployment one, in terms of the biological processes these represent. Whereas the pre-deployment network shows biological processes more related to an unexcited steady-state immune system, the post-deployment network displays several GO groups and GO terms which lie under the context of immunoenhancement. Reconstructed GNs thereby model two different situations in terms of the biological context. This also suggests the potential use of GNs for diagnostic purposes.

With regard to differential expression, a considerable gene up-regulation is observed, which correlates to immunoenhancement upon PTSD development. In general, the above mentioned GO terms are indicative of a nonspecific immune response, characteristic of innate immunity, suggesting the potential role of myeloid leukocytes in PTSD. Quite significant is also the GO group “hematopoietic or lymphoid organ development”, as the immune system is generated from multipotent hematopoietic stem cells, which branch in myeloid and lymphoid progenitors. This myeloid cell line comprises cells such as basophils, neutrophils, eosinophils and macrophages, which through immunosurveillance are responsible for the so-called unspecific or innate immunity. This is consistent with the results found by Breen et al. [42], who predicted the intrinsic role of innate immunity upon PTSD. These findings were also highlighted in a previous study by Watson et al. [62], who observed enhanced immunological features in PTSD-diagnosed Vietnam combat veterans in comparison with civilians.

## 4. Conclusions

In this paper, we introduce EnGNet, an ensemble-based novel method for the inference of large gene co-expression networks. First, EnGNet applies an ensemble approach for large co-expression networks reconstruction. Second, a greedy strategy optimizes both the size and topological features of the final network.

When compared with other standard approaches from the literature, EnGNet-inferred networks were smaller in size than those of other approaches, regarding the number of edges. In addition to achieving competitive results in terms of the presented biological information, EnGNet-inferred networks showed better performance in respect of networks topological, and thus biological, features. Among these features, sparseness and scale-free topology are to be highlighted as a major convenience of EnGNet networks, in concordance with actual GRN. In addition, EnGNet was demonstrated to be a competitive solution for studies on small datasets, by means of the experiments carried out. Moreover, topological features of EnGNet networks enable friendlier interpretation and hypothesis-making by life scientists.

Finally, the biological relevance of EnGNet was successfully tested in the application to human PTSD dataset. EnGNet inferred gene association networks from the gene expression dataset, revealing an innate immunity-mediated response in PTSD cases, which was accompanied by considerable gene upregulation. In particular, myeloid cells activation was detected in PTSD cases when compared to non-PTSD ones. Such PTSD-associated genes could then be considered as potential biomarkers, which can be used as pathology indicators. Besides, the GN inference approach distinguished between two different biological situations basing on gene expression, whereas analyses such as PCoA did not. These results demonstrate the usefulness of EnGNet in the field of biomarkers discovery, a field that has become one of the most relevant in personalized medicine.

## Figures and Tables

**Table 1:** Example representation of the major voting strategy to evaluate gene pairs.

Gene Pair	Spearman	Kendall	NMI	Final
$g_{1},g_{2}$	Correct	Correct	Correct	Correct
$g_{2},g_{3}$	Incorrect	Correct	Correct	Correct
$g_{4},g_{5}$	Incorrect	Correct	Incorrect	Incorrect
$g_{5},g_{2}$	Correct	Incorrect	Incorrect	Incorrect

**Table 2:** The results obtained by different gene networks on the yeast dataset using different thresholds. The precision and recall results were obtained using GeneMANIA database as gold standard. The last row presents the average results in terms of precision and size of the network for the experiment.

Thr		EnGNet	NMI	Kendall	Spearman	Ensemble
0.7	**Nodes**	3123	2684	2581	5371	3123
	**Rods**	7129	26,633	14771	455,776	33,715
	**Precision**	0.480	0.365	0.541	0.334	0.43
	**Recall**	0.002	0.01	0.009	0.041	0.01
0.8	**Nodes**	1057	1070	544	4180	620
	**Rods**	1296	4518	599	88,508	781
	**Precision**	0.555	0.416	0.773	0.412	0.514
	**Recall**	0.005	0.012	0.011	0.016	0.001
0.9	**Nodes**	258	1032	8	1375	258
	**Rods**	176	4398	4	3471	245
	**Precision**	0.657	0.409	1	0.639	0.651
	**Recall**	0.012	0.013	0.04	0.008	0.015
	**Avg. Precision**	0.56	0.39	**0.77**	0.46	0.53
	**Avg. Size**	2808.51	10,383.8	5123.59	181,428.13	11,498.83

**Table 3:** The results obtained by different gene networks on the Pre-deployment samples of the PTSD dataset using different thresholds. The precision and recall results were obtained using GeneMANIA database as gold standard. The last row presents the average results in terms of precision and size of the network for the experiment.

Thr		EnGNet	NMI	Kendall	Spearman	Ensemble
0.7	**Nodes**	1104	1026	941	5407	1098
	**Rods**	1222	9299	10,055	605,409	10,274
	**Precision**	0.407	0.112	0.294	0.138	0.294
	**Recall**	0.009	0.023	0.068	0.08	0.053
0.8	**Nodes**	131	823	98	2716	131
	**Rods**	110	8971	110	108,861	142
	**Precision**	0.635	0.112	0.611	0.195	0.633
	**Recall**	0.06	0.034	0.1	0.073	0.081
0.9	**Nodes**	5	775	0	624	5
	**Rods**	3	8943	0	4177	4
	**Precision**	1	0.112	0	0.301	1
	**Recall**	0.333	0.037	0	0.059	0.444
	**Avg. Precision**	**0.67**	0.11	0.30	0.21	0.64
	**Avg. Size**	462.66	9071	3388.33	239,482.33	3473.33

**Table 4:** The results obtained by different gene networks on the Human SNP dataset using different thresholds. The precision and recall results were obtained using GeneMANIA database as gold standard. The last row presents the average results in terms of precision and size of the network for the experiment.

Thr		EnGNet	NMI	Kendall	Spearman	Ensemble
0.7	**Nodes**	1553	259	1595	20,668	1544
	**Rods**	1963	202	5314	725,553	5049
	**Precision**	0.653	0.380	0.675	0.200	0.684
	**Recall**	0.020	0.023	0.043	0.022	0.044
0.8	**Nodes**	280	59	251	6853	241
	**Rods**	467	39	403	50309	381
	**Precision**	0.840	0.190	0.7607	0.398	0.771
	**Recall**	0.074	0.032	0.101	0.020	0.1120
0.9	**Nodes**	30	37	32	813	30
	**Rods**	16	26	25	2023	21
	**Precision**	0.6	0.15	0.5	0.727	0.428
	**Recall**	0.1875	0.0338	0.1818	0.0610	0.1875
	**Avg. Precision**	**0.69**	0.24	0.64	0.44	0.62
	**Avg. Size**	815.33	89	1914	259,295	1817

**Table 5:** The results obtained by different gene networks on the Post-deployment samples of the PTSD dataset using different thresholds. The precision and recall results were obtained using GeneMANIA database as gold standard. The last row presents the average results in terms of precision and size of the network for the experiment.

Thr		EnGNet	NMI	Kendall	Spearman	Ensemble
0.7	**Nodes**	1723	1303	1508	5958	1715
	**Rods**	2491	7381	37912	1718641	38641
	**Precision**	0.318	0.125	0.253	0.104	0.252
	**Recall**	0.006	0.012	0.091	0.147	0.075
0.8	**Nodes**	352	882	273	3516	351
	**Rods**	347	6479	753	325270	855
	**Precision**	0.456	0.119	0.522	0.155	0.503
	**Recall**	0.02	0.02	0.079	0.109	0.057
0.9	**Nodes**	9	750	4	982	9
	**Rods**	5	6375	2	14635	5
	**Precision**	1	0.116	1	0.294	1
	**Recall**	0.71	0.028	0.667	0.086	0.714
	**Avg. Precision**	**0.59**	0.12	0.59	0.18	0.58
	**Avg. Size**	947.66	6745	12889	686182	13167

**Table 6:** Yeast feature.

Thr		EnGNet	NMI	Kendall	Spearman	Ensemble
0.7	**Clust. Coef**	0.114	0.282	0.262	0.416	0.272
	**CPL**	7.201	6.947	5.406	2.978	4.358
	**Diameter**	29	28	22	10	20
	**Density**	0.001	0.007	0.004	0.032	0.007
	**Gamma**	1.413	0.958	1.529	0.915	1.286
0.8	**Clust. Coef**	0.283	0.524	0.162	0.342	0.163
	**CPL**	4.567	2.011	5.56	4.083	6.984
	**Diameter**	18	10	19	13	23
	**Density**	0.004	0.008	0.004	0.01	0.004
	**Gamma**	1.203	0.823	2.223	1.202	1.825
0.9	**Clust. Coef**	0.409	0.549	-	0.239	0.167
	**CPL**	2.401	1.007	1	6.726	2.57
	**Diameter**	6	2	1	24	7
	**Density**	0.007	0.008	0.143	0.004	0.007
	**Gamma**	0.934	0.66	-	1.782	1.981
Average	**Clust. Coef**	0.27	0.45	0.21	0.33	**0.20**
	**CPL**	**4.72**	3.32	3.99	4.60	4.64
	**Diameter**	**17.67**	13.33	14.00	15.67	16.67
	**Density**	**0.004**	0.008	0.050	0.015	0.01
	**Gamma**	1.18	0.81	**1.88**	1.30	1.70

**Table 7:** HUMANSNP.

Thr		EnGNet	NMI	Kendall	Spearman	Ensemble
0.7	**Clust. Coef**	0.055	0.119	0.235	0.219	0.239
	**CPL**	9.469	1.719	6.605	3.685	6.687
	**Diameter**	24	6	18	13	18
	**Density**	0.001	0.006	0.003	0.03	0.003
	**Gamma**	1.415	2.124	1.31	1.272	1.305
0.8	**Clust. Coef**	0.145	0.169	0.25	0.224	0.24
	**CPL**	2.543	1.026	2.551	5.231	2.573
	**Diameter**	8	2	8	23	7
	**Density**	0.007	0.022	0.009	0.02	0.009
	**Gamma**	1.01	1.98	1.447	1.374	1.486
0.9	**Clust. Coef**	0	0.27	0.073	0.238	0.1
	**CPL**	1.111	1.037	1.174	4.429	1.056
	**Diameter**	2	2	2	14	2
	**Density**	0.037	0.039	0.038	0.004	0.039
	**Gamma**	3.807	1.72	1.712	1.407	2.221
Average	**Clust. Coef**	**0.07**	0.19	0.19	0.23	0.19
	**CPL**	4.37	1.26	3.44	**4.45**	3.44
	**Diameter**	11.33	3.33	9.33	**16.67**	9.00
	**Density**	**0.015**	0.022	0.017	0.018	0.02
	**Gamma**	**2.08**	1.94	1.49	1.35	1.67

**Table 8:** Pre-deployment samples of the PTSD dataset.

Thr		EnGNet	NMI	Kendall	Spearman	Ensemble
0.7	**Clust. Coef**	0.031	0.689	0.499	0.615	0.444
	**CPL**	6.315	1.464	3.346	3.191	3.72
	**Diameter**	28	11	16	14	18
	**Density**	0.002	0.018	0.023	0.041	0.017
	**Gamma**	1.589	0.252	1.043	0.748	1.081
0.8	**Clust. Coef**	0.13	0.797	0.171	0.581	0.195
	**CPL**	2.859	1.001	2.721	3.485	2.785
	**Diameter**	9	3	6	15	8
	**Density**	0.012	0.027	0.023	0.03	0.017
	**Gamma**	1.426	0.142	1.571	0.847	1.681
0.9	**Clust. Coef**	0	0.843	0	0.449	0.6
	**CPL**	1.25	1	0	3.041	1
	**Diameter**	2	1	0	10	1
	**Density**	0.03	0.03	0	0.021	0.4
	**Gamma**	2	0.107	0	1.125	0.585
Average	**Clust. Coef**	**0.05**	0.78	0.22	0.55	0.41
	**CPL**	**3.47**	1.16	2.02	3.24	2.50
	**Diameter**	**13.00**	5.00	7.33	**13.00**	9.00
	**Density**	**0.01**	0.03	0.02	0.03	0.14
	**Gamma**	**1.67**	0.17	0.87	0.91	1.12

**Table 9:** Post-deployment samples of the PTSD dataset.

Thr		EnGNet	NMI	Kendall	Spearman	Ensemble
0.7	**Clust. Coef**	0.069	0.572	0.556	0.694	0.51
	**CPL**	5.207	7.142	3.17	2.592	3.309
	**Diameter**	17	22	12	13	12
	**Density**	0.002	0.009	0.033	0.097	0.026
	**Gamma**	1.301	0.862	0.859	0.507	0.763
0.8	**Clust. Coef**	0.245	0.706	0.344	0.632	0.295
	**CPL**	4.085	1.16	3.325	2.998	3.418
	**Diameter**	13	10	8	20	8
	**Density**	0.008	0.017	0.02	0.053	0.014
	**Gamma**	1.206	0.321	1.266	0.707	1.399
0.9	**Clust. Coef**	0	0.813	-	0.515	0
	**CPL**	1.167	1	1	3.523	1.167
	**Diameter**	2	1	1	13	2
	**Density**	0.139	0.023	0.333	0.03	0.139
	**Gamma**	3	0.218	-	0.954	3
Average	**Clust. Coef**	**0.10**	0.70	0.45	0.61	0.27
	**CPL**	**3.49**	3.10	2.50	3.04	2.63
	**Diameter**	10.67	11.00	7.00	**15.33**	7.33
	**Density**	**0.05**	0.02	0.13	0.06	0.06
	**Gamma**	**1.84**	0.47	1.06	0.72	1.72

**Table 10:** Average topological feature results for all methods in all datasets.

	EnGNet	NMI	Kendall	Spearman	Ensemble
**Clust. Coef**	**0.123**	0.528	0.268	0.430	0.269
**CPL**	**4.015**	2.210	2.988	3.830	3.302
**Diameter**	13.167	8.167	9.417	**15.167**	10.500
**Density**	**0.021**	0.018	0.053	0.031	0.057
**Gamma**	**1.692**	0.847	1.325	1.070	1.551

**Table 11:** Analysis to determine the $Th_{\beta }$ optimal value.

		*Th_β_* Values		
		**0.7**	**0.8**	**0.9**
**Pre**	**Nodes**	116	**105**	105
	**Rods**	119	**90**	87
	**Precision**	0.59	**0.63**	0.61
	**Recall**	0.07	**0.07**	0.06
**Post**	**Nodes**	437	298	**298**
	**Rods**	945	295	**272**
	**Precision**	0.313	0.481	**0.5**
	**Recall**	0.002	0.002	**0.002**

## Appendix A. PTSD Application Reconstructed Networks

Pre- and post-deployment networks, respectively, comprising 105 and 298 nodes, are shown in Figure A1. An increase is observed in the number of genes involved in post-deployment samples compared to pre-deployment ones. Such increase may well be the result of the genetic regulation upon PTSD that is addressed along Section 3.3. Gene FC is also represented in Figure A2, which revealed an overall genetic upregulation.

The union of the reconstructed networks is shown in Figure A2. Among the 310 genes comprised in this merged network, 229 showed an upregulation in the post-deployment situation compared to the pre-deployment samples.
